# Adjustment Disorder in Pregnant Women: Prevalence and Correlates in a Northern Mexican City

**DOI:** 10.14740/jocmr2275w

**Published:** 2015-08-23

**Authors:** Cosme Alvarado-Esquivel, Antonio Sifuentes-Alvarez, Carlos Salas-Martinez

**Affiliations:** a Biomedical Research Laboratory, Faculty of Medicine and Nutrition, Juarez University of Durango State, Durango, Mexico; b Mothers and Children’s Hospital, Secretary of Health, Durango, Mexico

**Keywords:** Adjustment disorder, Pregnancy, Epidemiology, Mexico

## Abstract

**Background:**

The epidemiology of adjustment disorder in pregnant women is largely unknown. We sought to determine the prevalence and correlates of adjustment disorder in pregnant women in Durango City, Mexico.

**Methods:**

Pregnant women (n = 300) attending in a public hospital in Durango City, Mexico were studied. All enrolled pregnant women had a psychiatric interview to evaluate the presence of adjustment disorder using the DSM-IV criteria. A questionnaire was submitted to obtain general epidemiological data of the pregnant women studied. Bivariate and multivariate analyses were used to assess the association of adjustment disorder with the epidemiological data of the women studied.

**Results:**

Fifteen (5.0%) of the 300 women studied had adjustment disorder according to the DSM-IV criteria. Adjustment disorder was not associated with age, occupation, marital status, or education of pregnant women. In contrast, multivariate analysis of socio-demographic, clinical and psychosocial variables showed that adjustment disorder was associated with the variables lack of support from her couple (odds ratio (OR) = 3.83; 95% confidence interval (CI): 1.00 - 14.63; P = 0.04) and couple living abroad (OR = 10.12; 95% CI: 1.56 - 65.50; P = 0.01).

**Conclusions:**

This is the first report about the epidemiology of adjustment disorder in pregnant women in Mexico. Results provide evidence of the presence of adjustment disorder and contributing psychosocial factors associated with this disorder in pregnant women in Mexico. Results point towards further clinical and research attention should be given to this neglected disorder in pregnant women.

## Introduction

Adjustment disorder is a common diagnosis in a number of clinical settings including primary care, general medical practice [1], and psychiatric practice [2]. However, adjustment disorder has received little attention in research settings [1-4]. Instead, scientific attention has been focused on major depression [5]. Adjustment disorder is a constellation of stress-related conditions or significant distress that occurs in response to exposure to a distressing event [6]. Adjustment disorder carries a significant rate of morbidity [2]. A stressor is the cause of the adjustment disorder, and its diagnosis is based on the longitudinal course of symptoms in the context of the stressor [7]. Adjustment disorders have been classified under the trauma and stress-related disorders recently [3]. Adjustment disorders are found in all cultures and ages [7]. The prevalence of this disorder varies among groups. A 2.94% prevalence of adjustment disorder has been found in patients from primary healthcare centers in Catalonia, Spain [8]. Whereas a 12.5% prevalence has been reported in cancer patients [9]. A high prevalence (36.5%) has been found in women referred to a consultation liaison psychiatric service because of positive scores on the Edinburgh postnatal depression scale in Queensland, Australia [10]. Treatment of adjustment disorders is psychotherapy [4, 11].

Very little is known about the epidemiology of adjustment disorders in pregnant women. To the best of our knowledge, there is not any report about the magnitude of these disorders in pregnant women in Mexico. Therefore, we sought to determine the prevalence and correlates of adjustment disorders in pregnant women in Durango City, Mexico.

## Materials and Methods

### Pregnant women studied and diagnosis of adjustment disorder

Three hundred pregnant women attending routine prenatal consultations in a public hospital (Mothers and Children’s Hospital of the Secretary of Health) in Durango City, Mexico were enrolled in the study. Women were selected by random sampling from January to December 2013. Inclusion criteria for enrollment in the study were: 1) pregnant women within their 1 - 9 months of pregnancy; and 2) who voluntarily accepted to participate. Age, socioeconomic status, educational level, and occupation were not restrictive criteria for enrollment. All women had a psychiatric consultation during pregnancy and diagnosis of adjustment disorder was based on the DSM-IV criteria [12].

### Epidemiological characteristics of the pregnant women

Socio-demographic, clinical and psychosocial characteristics of the participants were recorded in a questionnaire through a face-to-face interview (Supplementary 1, http://www.jocmr.org). Age, occupation, marital status, education, birthplace, residence, religion, having a health insurance, age at marriage, and number of marriages were obtained from all women studied. Clinical items included health status, obstetric history, gestational age, number of fetuses in the current pregnancy, fetal sex, and size and health status of the fetus. In addition, information about history of depression, stress or anxiety before or during pregnancy, smoking, consumption of alcohol, drug abuse, history of depression before pregnancy or trauma in life, history of complications during their last delivery, history of breastfeeding, health status of their last newborn, and number of children from all participants was obtained. Psychosocial items were: history of separation from parents at young age, presence of financial or family problems, bad relation with her mother in law, satisfaction with her education or body image, support from her couple, relatives, friends, colleagues or government, intended pregnancy, happiness for the sex of the fetus, bad relation with her couple, currently living with her couple, abandoned by her couple, violence from her couple, and couple living abroad.

### Statistical analysis

Data were analyzed with the aid of the software SPSS version 15.0. The association between adjustment disorder and characteristics of the pregnant women was assessed by bivariate and multivariate analyses. We used the Pearson’s Chi-square test and the Fisher exact test (when values were less than 5) for comparison of frequencies among groups. Multivariate analysis was performed only with variables with a P value equal to or less than 0.05 obtained in the bivariate analysis. Odd ratios (OR) and 95% confidence intervals (CIs) were calculated by logistic regression using the Enter method. Statistical significance was set at a P value < 0.05.

### Ethical aspects

Participants were informed about the aims and procedures of the study and a written informed consent was obtained from all them. The Ethical Committee of the Mothers and Children’s Hospital of the Secretary of Health in Durango City, Mexico approved this study.

## Results

Pregnant women studied were 23.39 ± 8.0 years old (range 13 - 45 years). They were examined for adjustment disorder once within their 2 - 9 months (median: 7 months) of pregnancy. Of the 300 pregnant women studied, 126 (42.0%) were primigravidae and 174 (58.0%) were multigravidae (2 - 8 pregnancies).

Fifteen (5.0%) of the 300 women studied had adjustment disorder according to the DSM-IV criteria. General socio-demographic characteristics of the pregnant women studied and their correlation with prevalence of adjustment disorder are shown in Table 1. The characteristic number of marriages was associated with adjustment disorder by bivariate analysis (P = 0.02). Other socio-demographic characteristics including age, occupation, marital status, education, birthplace, residence, religion, having a health insurance, and age at marriage did not show association with adjustment disorder (P < 0.05).

**Table 1:** Socio-Demographic Characteristics of the Pregnant Women and Their Association With Adjustment Disorder

Characteristics	No. of women studied	Adjustment disorder		P value
		No.	%	
Age (years)				
13 - 17	116	4	3.4	0.32
> 17	184	11	6	
Occupation				
Laborer	19	1	5.3	1.00
Non-laborer	281	14	5	
Marital status				
Married	91	3	3.3	0.44
Single	48	5	10.4	
Divorced	1	0	0	
Living together	159	7	4.4	
Widowed	1	0	0	
Education (years)				
None	1	0	0	0.06
1 - 6 years	36	5	13.9	
7 - 12 years	251	9	3.6	
> 12 years	12	1	8.3	
Birthplace				
Durango State	299	15	5	1.00
Abroad	1	0	0	
Residence place				
Durango State	297	15	5.1	1.00
Other Mexican State	3	0	0	
Residence area				
Urban	249	10	4	0.22
Suburban	19	2	10.5	
Rural	32	3	9.4	
Religion				
Yes	289	15	5.2	1.00
No	11	0	0	
Health insurance				
Yes	297	15	5.1	1.00
No	3	0	0	
Age at marriage				
13 - 17	163	5	3.1	0.37
> 17	119	7	5.9	
No. of marriages				
None	16	3	18.8	0.02
One	240	9	3.8	
More than one	44	3	6.8	

Of the clinical characteristics of the pregnant women studied, the variables trauma in life and depression during pregnancy were associated with adjustment disorder by bivariate analysis. Other clinical characteristics including health status, obstetric history, gestational age, number of fetuses in the current pregnancy, fetal sex, size and health status of the fetus, history of stress or anxiety before or during pregnancy, smoking, consumption of alcohol, drug abuse, history of depression before pregnancy, history of complications during their last delivery, history of breastfeeding, health status of their last newborn, and number of children did not show association with adjustment disorder by bivariate analysis. Table 2 shows a correlation between a selection of clinical variables and prevalence of adjustment disorder in the pregnant women studied. Women with adjustment disorder were treated with psychotherapy.

**Table 2:** Results of the Bivariate Analysis of a Selection of Clinical Characteristics of the Pregnant Women and Their Association With Adjustment Disorder

Characteristics	No. of women studied	Adjustment disorder		P value
		No.	%	
Trimester of pregnancy				
First	16	0	0	0.41
Second	87	3	3.4	
Third	197	12	6.1	
Depression during pregnancy				
Yes	102	9	8.8	0.02
No	198	6	3	
Stress during pregnancy				
Yes	135	9	6.7	0.23
No	164	6	3.7	
Fetal health status				
Healthy	294	14	4.8	0.14
Ill	3	1	33.3	
Outcome of last pregnancy				
Delivery	57	1	1.8	0.30
Cesarean section	82	6	7.3	
Miscarriage	3	0	0	
Health status of last newborn				
Healthy	132	6	4.5	0.30
Ill	15	1	6.7	
Dead	5	1	20	
Depression before pregnancy				
Yes	97	8	8.2	0.09
No	202	7	3.5	
Trauma in life				
Yes	51	6	11.8	0.02
No	248	9	3.6	
Stress before pregnancy				
Yes	102	7	6.9	0.28
No	198	8	4	

With respect to psychosocial characteristics, the variables lack of support from her couple and couple living abroad were associated with adjustment disorder by bivariate analysis. Other psychosocial variables including history of separation from parents at young age, presence of financial or family problems, bad relation with her mother in law, satisfaction with her education or body image, support from her relatives, friends, colleagues or government, intended pregnancy, happiness for the sex of the fetus, bad relation with her couple, currently living with her couple, abandoned by her couple, and violence from her couple did not show association with adjustment disorder. A selection of psychosocial characteristics of the pregnant women studied and their association with prevalence of adjustment disorder is shown in Table 3.

**Table 3:** Results of the Bivariate Analysis of a Selection of Psychosocial Characteristics of the Pregnant Women and Their Association With Adjustment Disorder

Characteristics	No. of women studied	Adjustment disorder		P value
		No.	%	
Separated from parents at young age				
Yes	94	7	7.4	0.25
No	206	8	3.9	
Financial problems				
Yes	115	9	7.8	0.07
No	185	6	3.2	
Family problems				
Yes	37	3	8.1	0.40
No	263	12	4.6	
Satisfaction with educational level				
Yes	167	10	6	0.37
No	133	5	3.8	
Support from her couple				
Yes	262	8	3.1	0.003
No	37	6	16.2	
Desired pregnancy				
Yes	192	8	4.2	0.37
No	108	7	6.5	
Relation with her couple				
Good	249	9	3.6	0.23
Bad	48	4	8.3	
Living with her couple				
Yes	238	9	3.8	0.28
No	57	4	7	
Ever abandoned by her couple				
Yes	91	6	6.6	0.22
No	205	7	3.4	
Violence from her couple				
Yes	47	2	4.3	1.00
No	249	11	4.4	
Couple living abroad				
Yes	8	2	25	0.04
No	286	11	3.8	

Multivariate analysis of socio-demographic, clinical and psychosocial variables with P values < 0.05 obtained by bivariate analysis showed that adjustment disorder was only associated with the variables lack of support from her couple (OR = 3.83; 95% CI: 1.00 - 14.63; P = 0.04) and couple living abroad (OR = 10.12; 95% CI: 1.56 - 65.50; P = 0.01). Table 4 shows results of the multivariate analysis.

**Table 4:** Multivariate Analysis of Selected Characteristics of Pregnant Women and Their Association With Adjustment Disorder

Characteristic	Odds ratio	95% confidence interval	P value
Number of marriages	0.51	0.15 - 1.66	0.26
Trauma in life	2.41	0.63 - 9.23	0.19
Depression during pregnancy	1.00	0.26 - 3.84	0.98
Lack of support from her couple	3.83	1.00 - 14.63	0.04
Couple living abroad	10.12	1.56 - 65.50	0.01

## Discussion

The epidemiology of adjustment disorder has been poorly studied in Mexico in general, and there is a lack of information about the epidemiology of this disorder in pregnant women in this country in particular. The present study aimed to determine the prevalence and correlates of adjustment disorder in a sample of pregnant women in the northern Mexican city of Durango. We found a 5.0% prevalence of adjustment disorder in the pregnant women studied. Comparison of the prevalence of adjustment disorder found in our study with those found in other studies can be hardly performed because there are only few studies about the prevalence of adjustment disorder in population groups reported in the medical literature. This fact is consistent with the reportedly poor attention that this disorder has received in research settings [1-4]. The prevalence of adjustment disorder found in pregnant women in our study is comparable with a 2.94% prevalence of adjustment disorder reported in patients from primary healthcare centers in Catalonia, Spain [8]. In contrast, the prevalence of adjustment disorder found in pregnant women in Durango City is lower than a 12.5% prevalence found in cancer patients in Leipzig, Germany [9]. Similarly, the prevalence found in our study is lower than a 36.5% prevalence found in women referred to a consultation liaison psychiatric service because of positive scores on the Edinburgh postnatal depression scale in Queensland, Australia [10]. The lower prevalence of adjustment disorder in pregnant women in our study than those reported in cancer patients and women referred to psychiatric service can be due to differences in health status among groups. We included mostly healthy women in our study whereas the other studies included ill participants or probably suffering from depression since they had positive scores for depression on the Edinburgh postnatal depression scale.

We searched for contributing factors associated with adjustment disorder in pregnant women and multivariate analysis showed that this disorder was associated with a lack of support from her couple and couple living abroad. To the best of our knowledge, this is the first report about the association of these factors with adjustment disorder. There is a lack of reports about contributing factors for adjustment disorder in pregnant women; therefore, we are unable to compare the contributing factors found in the present study with those in other studies.

The present study has a limitation; pregnant women studied were enrolled in one public hospital. The participating hospital attends mostly people of low socioeconomic level. Therefore, it is unclear whether pregnant women attending private hospitals or belonging to a medium or high socioeconomic level might have the same prevalence of adjustment disorder or the contributing factors as those found in the pregnant women studied.

### Conclusions

This is the first report about the epidemiology of adjustment disorder in pregnant women in Mexico. Our results provide evidence of the presence of adjustment disorder and contributing psychosocial factors associated with this disorder in pregnant women in Durango, Mexico. Results point towards further clinical and research attention should be given to the adjustment disorder in pregnant women.

## References

[1] Casey P (2001). Adult adjustment disorder: a review of its current diagnostic status. J Psychiatr Pract.

[2] Patra BN, Sarkar S (2013). Adjustment disorder: current diagnostic status. Indian J Psychol Med.

[3] Casey P (2014). Adjustment disorder: new developments. Curr Psychiatry Rep.

[4] Carta MG, Balestrieri M, Murru A, Hardoy MC (2009). Adjustment Disorder: epidemiology, diagnosis and treatment. Clin Pract Epidemiol Ment Health.

[5] Casey P (2006). The 'afterthought' diagnosis: rehabilitating adjustment disorders. Expert Rev Neurother.

[6] Akutsu PD, Abhari B (2014). Adjustment disorders in Asians and Asian Americans. Asian J Psychiatr.

[7] Casey P (2009). Adjustment disorder: epidemiology, diagnosis and treatment. CNS Drugs.

[8] Fernandez A, Mendive JM, Salvador-Carulla L, Rubio-Valera M, Luciano JV, Pinto-Meza A, Haro JM (2012). Adjustment disorders in primary care: prevalence, recognition and use of services. Br J Psychiatry.

[9] Mehnert A, Vehling S, Scheffold K, Ladehoff N, Schon G, Wegscheider K, Heckl U (2013). [Prevalence of adjustment disorder, acute and posttraumatic stress disorders as well as somatoform disorders in cancer patients]. Psychother Psychosom Med Psychol.

[10] Harvey ST, Pun PK (2007). Analysis of positive Edinburgh depression scale referrals to a consultation liaison psychiatry service in a two-year period. Int J Ment Health Nurs.

[11] Laugharne J, van der Watt G, Janca A (2009). It is too early for adjusting the adjustment disorder category. Curr Opin Psychiatry.

[12] American Psychiatric Association Diagnostic and Statistical Manual of Mental Disorders: DSM-IV-TR, 4th ed. Washington, D.C. 2000.

